# Genotype 1 Hepatitis C Virus Envelope Features That Determine Antiviral Response Assessed through Optimal Covariance Networks

**DOI:** 10.1371/journal.pone.0067254

**Published:** 2013-06-20

**Authors:** John M. Murray, Rémy Moenne-Loccoz, Aurélie Velay, François Habersetzer, Michel Doffoël, Jean-Pierre Gut, Isabel Fofana, Mirjam B. Zeisel, Françoise Stoll-Keller, Thomas F. Baumert, Evelyne Schvoerer

**Affiliations:** 1 School of Mathematics and Statistics, University of New South Wales, Sydney, NSW, Australia; 2 The Kirby Institute, University of New South Wales, Sydney, NSW, Australia; 3 Pôle Hépato-digestif, Hôpitaux Universitaires de Strasbourg, Nouvel Hôpital Civil, Strasbourg, France; 4 Université de Lorraine, EA Stress Immunité et Pathogènes, Vandoeuvre-les-Nancy, France; 5 CHU Nancy, Pôle Laboratoires, Virologie, Vandoeuvre-les-Nancy, France; 6 Institut National de la Santé et de la Recherche Médicale (Inserm), U748, Strasbourg, France; 7 Université de Strasbourg, Strasbourg, France; 8 Laboratoire de Virologie, Hôpitaux Universitaires de Strasbourg, Strasbourg, France; University of Hawaii Manoa, United States of America

## Abstract

The poor response to the combined antiviral therapy of pegylated alfa-interferon and ribavarin for hepatitis C virus (HCV) infection may be linked to mutations in the viral envelope gene E1E2 (*env*), which can result in escape from the immune response and higher efficacy of viral entry. Mutations that result in failure of therapy most likely require compensatory mutations to achieve sufficient change in envelope structure and function. Compensatory mutations were investigated by determining positions in the E1E2 gene where amino acids (aa) covaried across groups of individuals. We assessed networks of covarying positions in E1E2 sequences that differentiated sustained virological response (SVR) from non-response (NR) in 43 genotype 1a (17 SVR), and 49 genotype 1b (25 SVR) chronically HCV-infected individuals. Binary integer programming over covariance networks was used to extract aa combinations that differed between response groups. Genotype 1a E1E2 sequences exhibited higher degrees of covariance and clustered into 3 main groups while 1b sequences exhibited no clustering. Between 5 and 9 aa pairs were required to separate SVR from NR in each genotype. aa in hypervariable region 1 were 6 times more likely than chance to occur in the optimal networks. The pair 531–626 (EI) appeared frequently in the optimal networks and was present in 6 of 9 NR in one of the 1a clusters. The most frequent pairs representing SVR were 431–481 (EE), 500–522 (QA) in 1a, and 407–434 (AQ) in 1b. Optimal networks based on covarying aa pairs in HCV envelope can indicate features that are associated with failure or success to antiviral therapy.

## Introduction

Poor response rates for hepatitis C virus (HCV) infection are obtained by current combination antiviral therapy with pegylated alfa-interferon (IFN-α) and ribavarin (RBV) where only 45% of individuals with genotype 1 experience a sustained virological response (SVR, undetectable HCV RNA 6 months after the end of therapy). Success in therapy is determined by a number of host and viral factors including the patient's adherence to therapy, IL28B genotype, viral load and genotype [1]–[5]. The viral sequence can certainly affect the success rate of therapy since genotype 1 has poorer rates of SVR and even within that genotype, patients with 1a virus are less successful than patients with 1b virus [6]. For treatment that includes interferon, a drug that stimulates antiviral cytokines, failure of therapy for immunological reasons is even more likely determined by the viral sequence. New anti-HCV drugs such as the protease inhibitors telaprevir and boceprevir are now available for HCV genotype 1 treatment, but they are administered in tritherapy with IFN-alfa and RBV, which remains the backbone of anti-HCV treatment. Although coadministration of protease inhibitors improves SVR rates, treatment failure due to viral resistance and adverse events remain important challenges [7], [8].

It has been demonstrated that evasion from the immune response is linked to mutations in the envelope gene. The E1E2 region of the HCV genome (*env*) codes for the envelope E1 and E2 glycoproteins on the surface of virions, that are crucial in binding to target cells and are also themselves targets for the immune response. Mutations in this region lead to escape from neutralizing antibody and T cell responses [9], but can also affect infectivity [10]–[12]. Viral entry into hepatocytes is mediated by a number of host cell factors that link with the E1E2 glycoproteins. The hypervariable region 1 (HVR1) of E2 interacts with scavenger receptor BI (SR-BI) and highly sulfated heparin sulphate (HS) [13]. CD81 binds to a number of domains within E2 [14]. Mutations within this region can therefore be expected to have an impact on success of antiviral therapy [15].

Investigation of the impact of mutations in envelope can be complicated due to the high variability of this gene and the resulting difficulty in determining which mutations produce a functionally different phenotype. Our group has recently assessed the influence of single mutations from HCV *env* sequences on antiviral response through a combined bioinformatics and *in vitro* analysis. By constructing HCV pseudoparticles that expressed an alanine (A) at position 431, or a valine (V) at position 642, we were able to verify these 1a mutations, identified in an initial bioinformatics analysis, significantly decreased antibody neutralization, while the 431A mutation also increased entry of virus into Huh7.5 cells that over-expressed CD81 and SR-B1 [16]. However bioinformatics analyses by themselves have less power to determine single aa predictors of response, given any cohort usually includes considerably fewer patient sequences than the 555 aa in the envelope sequences.

Networks of positions in E1E2 where the aa covary across groups of patients likely indicate a connection between the aa that are required for function. The set of all covarying aa pairs offers a more robust basis upon which to extract information within E1E2 that i) is relevant to function, ii) that may be used as a means of predicting response, and iii) that can implicate regions instrumental in HCV-related mechanisms of treatment failure. This method of determining relatedness in genes has been used previously [17]–[20], as well as in the context of HCV [21]. Networks that use the covarying pairs as building blocks can generate sets that contain the many compensatory mutations required for a different functional response.

However sets of covarying pairs can be large and can also provide many choices for separation between response groups. Hence we investigated methods that included as few pairs as possible in the set associated with response, reasoning that such sets would contain the most important individual features, and also point to biologically relevant positions. We therefore determined minimal sets of aa pairs that could successfully separate good responders (SVR) from poor responders (NR). These minimal separation methods were investigated on pretreatment E1E2 sequences obtained from 92 individuals infected with HCV genotypes 1a or 1b, and receiving therapy of IFN-α and RBV [16].

## Methods

### Patients

Pre-treatment serum samples were collected from 92 patients infected by HCV genotype 1 (1a, n = 43; 1b, n = 49) followed at the hospitals: Centre Hospitalier-Universitaire (CHU) Strasbourg n = 44; CHU Tours n = 7; CHU Clermont-Ferrand n = 3; CHU Bobigny n = 16; CHU Villejuif n = 6; CHU Toulouse n = 5; Hôpital Pitié-Salpêtrière, Paris n = 5; CHU Bordeaux n = 4; CHU Brest n = 2. Approval of the study was obtained by the “Comité de Protection des Personnes – CPP d'Alsace” (19/11/2008, DC-2008-829), in accordance with the ethical guidelines of Helsinki. The study was realized on a sample collection performed in the context of classical viro-clinical follow-up by physicians who take care of their patients suffering from chronic hepatitis C. The physicians informed their patients that remaining blood sample volumes could be used for research on HCV treatment. They have then checked the verbal non-opposition from their patients who were given the information on the viro-clinical study (file available on request). Indeed, verbal non-opposition is convenient in this context (checked in 2008 with Clinical Research Authorities, University Hospital, Strasbourg). The local ethical authorities “Comité de Protection des Personnes – EST IV”, approved this sample collection in 2010 (file available on request).

### Alignment

Sequences were aligned with the reference strains H77 (1a) and J4 (1b) using a progressive multiple alignment method (multialign, Matlab 2010b). Pairwise distances were calculated with the Jukes-Cantor method, with the phylogenetic tree generated using the Unweighted Pair Group Method Average (seqlinkage).

### Calculation of covariance values 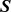


An 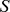 covariance value was determined for each aa pair position based on the method of Aurora et al. [21]. These calculations were performed separately for each genotype, as well as for each of the SVR and NR groups within these genotypes. We then extracted the covarying pairs above a background cut-off value 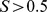 [21].

### Networks

We calculated sets of covarying pairs 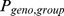, for each genotype (a or b), and where the calculations were performed over the group of all individuals within that genotype or separately for NR or SVR (All, NR, SVR). These sets determined initial networks where the aa formed the nodes, and edges connected aa that appeared in the set of covarying pairs.

Our aim was to calculate minimal networks based on each of these sets where we extract covarying pairs and particular aa combinations for that pair where a feature is present in one response group but not the other. For example aa at positions 373 and 438 might be covarying with 8 NR patients exhibiting a V at position 373 and a D at 438 while no SVR patients exhibit this feature of the VD combination. We term such pairs and aa combinations as *separating pairs*. Calculations of minimal networks were relevant to all possible separating pairs and so our first step was to construct the network of these. The networks for each of the analyses were constructed as outlined in the following example, which demonstrates the process of selecting aa combinations exhibited by one response group and not the other, in this case NR and not by SVR, from the set of covarying pairs in the genotype 1a SVR individuals 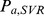.

#### Example

From the set of covarying pairs determined on the 1a SVR sequences 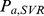, we extract aa combinations over these pairs that are exhibited by sequences of the NR individuals but not by the SVR individuals in genotype 1a. The set 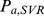 consists of 681 pairs listed by their positions within E1E2: [(7,28), (7,46), (7,139), …,(527,537), (527,548), (534,550)] involving 85 different positions [7,25,26,…,537,548,555]. Of these 681 pairs only 561 exhibit separating pairs (at least one aa combination appearing in NR sequences and not in SVR sequences). For instance at the positions (7,139) there are 5 NR individuals expressing the combination SA (a Serine at position 7 and an Alanine at position 139), while no SVR sequences exhibit this combination for this pair of positions. The combination SA at position (7,139) is therefore a separating pair. This combination was the only one unique to NR for this covarying pair while other covarying pairs exhibited between 0 and 16 separating pairs since there can be more than one aa combination at a given pair of positions that are exhibited by NR but not by SVR individuals. Over all covarying pairs in 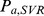 there were a total of 1,631 separating pairs.

The nodes of the network for this example are provided by the subset of positions where a separating aa pair is incident. For this example all 85 positions have at least one separating pair incident to them and therefore form the nodes of the network.

Separation between the response groups can be determined based on single separating aa pairs, or the entire collection of separating pairs for the given positions. Accordingly the edges in the network can be formed by single connections between aa pairs where any separating pair exists if we allow multiple features in the separation for each position, or with edges for each of the separating aa pairs if we only allow a single separating aa pair to be specified at each position. For example there are two separating aa pairs for (7,207), TG and SG. If we allow multiple features at each position in the separation then we include a single edge between nodes 7 and 207 representing the 3 individuals with a TG or SG at these positions. On the other hand if we only allow a single feature to be selected at each position then we include an edge representing the single individual with TG, and another edge representing the 2 individuals with SG. Calculation of a minimal network in the latter case includes constraints that only allow the choice of a single edge between nodes.

### Binary integer programming formulation of the problem of choosing optimal pairs from networks

Constructing the network where there are edges between nodes for each separating pair produces the coefficient matrix 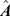, while representing all separating aa pairs between two nodes by a single edge produces the coefficient matrix 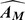. These networks and corresponding coefficient matrices were produced for each of the 12 individual analyses.

These 0–1 coefficient matrices contain a row for each individual (in NR for networks describing Poor Response and in SVR for networks describing Good Response), and a column or columns for the separating pairs at each covarying pair. This procedure is described as follows, for construction of coefficient matrices for Poor Response.

Suppose there are 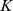 patients in NR and 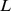 in SVR. We are also given a set of aa pairs 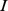 with indices 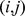 where these represent the pair positions in the viral sequence of the aa pair and where 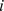 is the smallest index. For each index in 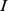 we determine the number of different amino acid combinations for positions 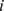 and 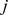 that appear in NR but not in SVR, 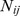. Let us denote this ordered set of pairs at these positions for patients in NR that do not appear in SVR as separating pairs at 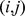. We then calculate the patient-pair matrix 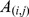 with dimensions 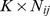 where the 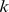 ^th^ row and 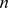 ^th^ column is 1 if the 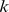 ^th^ patient in NR has the 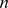 ^th^ separating pair and 0 otherwise.

For example suppose there are 5 patients in NR and 6 in SVR and at positions 1 and 2 in the amino acid sequence the patients exhibit the aa pairs shown in Table 1. Then the aa pairs that appear in NR but not in SVR are VS and AT. We let VS represent the first of these and AT the second. The patient-pair matrix is then given by
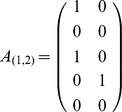



**Table 1:** Example of separating pairs. aa pairs at positions 1 and 2.

Response	Patient	aa at posn 1	aa at posn 2
**NR**	1	V	S
	2	A	Y
	3	V	S
	4	A	T
	5	A	S
**SVR**	6	A	S
	7	V	Y
	8	V	Y
	9	A	Y
	10	A	S
	11	A	Y

Patients 1 and 3 exhibited the first of these pairs so in the first and third row there is a 1 in the 1st column. Patient 4 exhibited the second of these pairs so in the 4th row there is a 1 in the second column. All other entries are zero.

If we do not need to differentiate the different separating pairs we can collapse each of these matrices into a single column by using instead the matrix 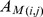 that consists of the sum of the above matrix over each row. This will also be a 0-1 matrix. 
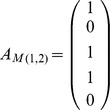



For all pairs in the set 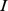 we construct the concatenated matrix of these individual separating matrices
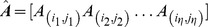



Similarly we obtain the concatenated matrix 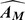.

We wish to determine a set of aa positional pairs 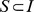 that exhibit the property such that for each patient in NR there is a separating aa pair for this patient at some position (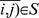. This constraint can be written as 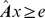
where 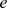 is the vector of 1's and 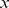 is a 0–1 column vector with the same number of components as columns in the matrix 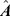 , if only a single feature is to be chosen in each aa pair. We will need to apply an additional constraint to ensure that only one of the columns from the individual 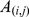 matrices corresponds to a 1 in the vector 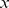 so that a single feature is chosen for each possible pair. If several features can be chosen to separate the sets in each pair, then this set of constraints is written as



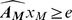



Some of the individual matrices 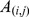 will be empty as no feature will separate the two sets at that pair. Therefore the columns of the concatenated matrix 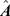 need not correspond in a one-to-one fashion with the ordered set of pairs 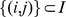.

Given a weight vector 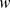 for the inclusion of each separating pair we obtain the binary programming problem described below. Binary integer programs are a standard operation research method of optimally choosing items from a set while satisfying constraints [22].
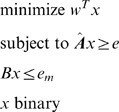
(1)


The matrix 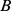 has rows corresponding to the number of 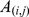 matrices that have more than one column. The elements of the 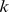 ^th^ row of 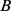 are zero except where the columns correspond to the 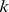 ^th^ matrix 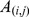 with multiple columns where it has values 1. This constraint ensures only one of these columns can be chosen.

If multiple features can be chosen then the problem becomes
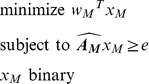
(2)


### Different weight vectors

If *w_k_* = 1, for all *k* then minimization determines sets that use the fewest number of pairs. Calculations of networks that maximized a measure of total covariance, while keeping numbers of pairs small, were performed through minimization with the weights
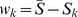
where 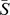 is the integer ceiling of the maximum of all covariance values 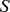. This choice ensures the weights are nonnegative, the minimization will preferentially include pairs with high 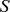, and will penalize the addition of pairs in general.

All calculations were performed with Matlab version R2010b (The MathWorks Inc., USA). The binary integer programming problems were solved using the bintprog routine.

## Results

**Figure 1:**
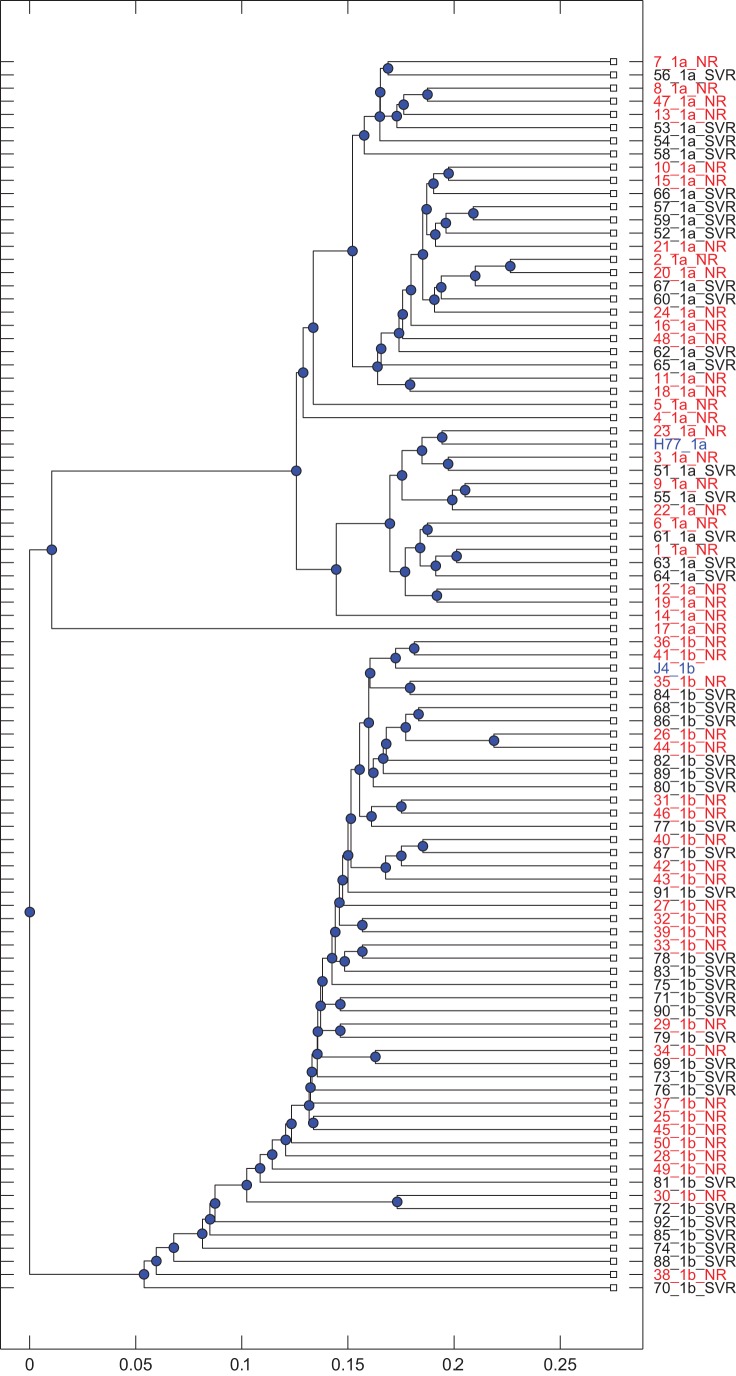
Phylogenetic tree of all individuals. Identifiers for nonresponders are coloured red. Reference strains H77 for 1a and J4 for 1b are also shown (blue).

We investigated the response to antiviral therapy with IFN-α and RBV, median duration of 9.9 months (range 6.4 to 13.4 months), in 92 HCV chronically infected individuals, 43 of whom were infected with genotype 1a and 49 with genotype 1b. Within genotype 1a, 17 individuals (40%) achieved SVR while there were 25 SVR individuals (51%) in genotype 1b. The E1E2 sequences comprising approximately 555 amino acids, enabled separation of genotypes phylogenetically as displayed in Figure 1. SVR individuals were scattered through the phylogenetic tree for each of the genotypes.

For each of the genotypes we calculated covarying aa pairs over sequences in each group (Methods). Calculations over all individuals in genotype 1a resulted in 1,095 pairs and 516 pairs in genotype 1b, considerably smaller than the total number of possible pairs (555×554/2 = 153,735). The individual positions in E1E2 contained in these covarying positions were largely consistent with those found previously in an analysis over the whole HCV genome. Positions within our covarying pair calculations exhibited a 79% overlap with those in E1E2 determined by Aurora et al. [21], while 83% of the Aurora positions also appeared in our calculations. Comparison of covarying pairs however showed a substantially smaller overlap: 21% of our pairs were in the Aurora calculations while 28% of their pairs appeared in our calculations.

### Conserved and covarying regions for each genotype

**Figure 2:**
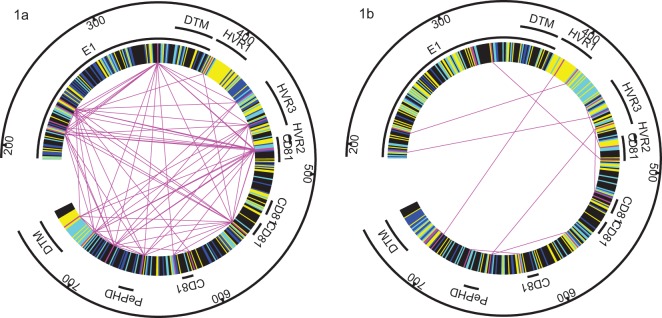
Conserved and covarying regions for 1a and 1b genotypes. Regions are coloured as conserved across both genotypes (black), conserved within each genotype (dark blue), conserved except for a maximum of 2 individuals in that genotype (light blue), and the positions within 50% of maximum covariance in each genotype (magenta) with the covarying pairs shown connected through magenta lines. Regions indicated: E1, transmembrane domains (DTM), hypervariable regions (HVR1, HVR2, HVR3), CD81 binding sites (CD81), and PePHD.

Higher covariance between positions suggests that these positions are functionally or structurally important for HCV. These positions are therefore more relevant in that sense than positions that are highly variable, but less relevant than conserved positions. We mapped regions that were conserved, covarying and variable in E1E2 over both genotypes and depicted them in Figure 2. We note that the regions that are conserved separately in genotypes (dark blue) appear in different regions of E1E2, although two positions 275 (L in 1a, M in 1b) and 362 (F in 1a, Y in 1b) were conserved differently in each genotype. Although the final 6 of the 28 aa in the transmembrane domain (DTM) for the E2 sequences were conserved by both genotypes, an additional 13 aa were conserved in 1b sequences with no additional 1a conserved positions.

**Figure 3:**
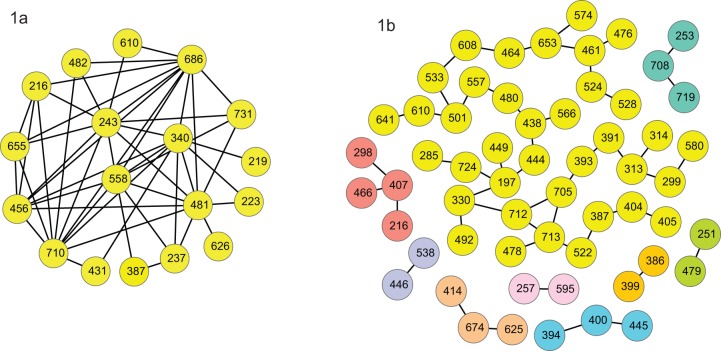
Networks of highest covarying pairs. The 50 aa pairs with the highest covariance values calculated over all individuals in genotypes a and b. Edges between nodes connect the covarying aa pairs.

Genotype 1a groups exhibited higher covariance values than their 1b counterparts. The 50 highest covariance pairs were also much more connected in 1a than 1b (Figure 3). These pairs for each genotype overlap at position 216, and are close at 480(b), 481(a), 482(a) and 625(b), 626(a) and 653(b), 655(a) and 710(a), 712(b), 713(b). Positions that were directly connected in the network for 1a showed similar subsequences for one subgroup. For example, position 216 is directly connected to positions 243, 456, 655, 686 and 710 in the network in Figure 3. Twelve individuals exhibited the sequence ATLESA at these positions with this exact sequence for 10 of the 12 with 1 aa variation for the remaining two individuals, compared to the sequence TTM(E/A)SA for 2 individuals, TAMFTV for 28 individuals, and TAMAT- for the remaining individual. The 14 individuals with the ATLESA or TTM(E/A)SA sequences form the bottom cluster in the phylogenetic tree for 1a. On the other hand the top cluster of 8 individuals exhibits a gap at position 731 within DTM for E2, whereas all but 2 others do not. In summary, covarying networks, especially for genotype 1a, can extract patients in different clusters and determine those aa positions that typify those clusters. This supports our hypothesis that networks of covarying pairs may also provide a means to differentiate treatment response groups, and that covarying networks can determine such features that may be specific to an individual cluster.

### Extracting optimal covarying networks that are associated with response

Antiviral response groups cannot be separated on conserved positions, nor will highly variable regions, by their very nature, provide a robust means of predicting response. Hence we followed the approach by Aurora et al. [21] who used covarying positions over the entire HCV genome to form networks that were exhibited by one response group but not the other. We hypothesized that determining networks of covarying pairs that separated response groups and satisfied certain optimality criteria might determine the most relevant of these pairs, and hence provide the most robust associations with response and point to the underlying process in the HCV structure by which this occurs.

Optimal networks were determined based on whether a single or multiple aa combinations were allowed at each separating pair, whether the criteria was to minimize the number of pairs or to maximize a measure of the total covariance, and whether we extract features in the NR (Poor Response) or the SVR (Good Response) groups. The covarying pairs can also be determined over all individuals in a genotype, or from the SVR or NR patient sequences. This led to 48 individual calculations and optimal networks (Supporting Information Table S1).

Incorporating the covariance value into the optimization criterion made little impact on the number of separating pairs in the optimal network compared to the optimization criterion that explicitly minimized the number of pairs, mostly due to both criteria penalizing separating networks with high numbers of edges. Combining the results for both of these criteria, the six optimal networks for poor response allowing multiple aa combinations at each pair only required 3 pairs in genotype 1a (Table S1). Optimal genotype 1b networks required between 2 and 4 pairs. Only allowing single combinations understandably increased the size of the optimal networks, ranging between 5 and 9 pairs.

**Figure 4:**
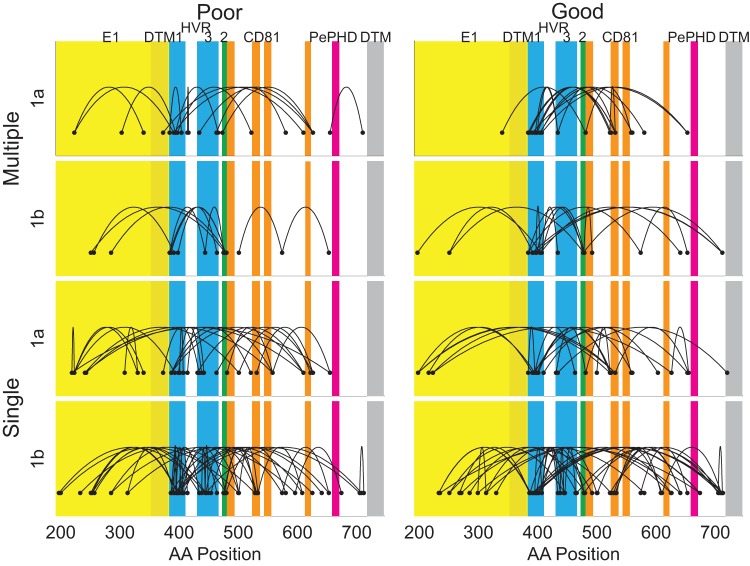
Mapping of optimal covarying pairs. Collections of all separating pairs in the minimal networks for each problem where selection can be within NR (Poor) or SVR (Good), for genotypes 1a and 1b, and where either only a single separating pair is chosen (Single), or multiple aa combinations are allowed for each pair (Multiple). The E1E2 sequence that ranges from aa positions of 192 to 746 is shown separated into E1 (yellow) with its transmembrane domain (DTM, darker yellow), HVR 1 and 3 (blue), with HVR2 that overlaps part of the CD81 binding site (green), the CD81 binding sites (CD81, orange and green where it overlaps with HVR2), PePHD (magenta), and the transmembrane domain for E2 (grey) [23], [26].

E1 covered 192 out of the 555 aa positions (35%) yet these appeared less frequently in optimal networks (Figure 4). For multiple combinations: Poor Response (NR), 6 E1 positions appeared among all 36 possible positions (twice the sum of the total number of pairs in these 6 optimal networks, 17%) in 1a, and 3 of 30 (10%) in 1b; Good Response (SVR), 3% in 1a, and 8% for 1b. The frequency of E1 positions was higher when optimal networks for single combinations were considered but was still lower than the expected frequency of 35% under random choice: Poor Response, 23% in 1a, and 14% in 1b; Good Response, 10% in 1a, and 16% for 1b.

Allowing multiple aa combinations for each pair in the network tended to result in pairs that contained a position within hypervariable region 1 (HVR1, positions 384–410, 5% of E1E2, Figure 4, [23]–[25]). For example each of the 3 pairs in the optimal network for Good Response, 1a, where we minimized the number of pairs and the separating set was SVR, contained a position within HVR1 (396, 399, 407, Table S1). The pair 396–434 for that network separated 6 SVR individuals but used 5 different aa combinations (AH, AT, LK, TS, VD). The inherent variability of positions within HVR1 resulted in many different separating pairs. However this was not always the case: 399–524 separated 9 SVR individuals based only on two aa combinations IA and LV, while 407–653 separated 4 SVR individuals with the single combination PN. For multiple combinations: Poor Response, HVR1 appeared in 36% of optimal networks for 1a, and 27% for 1b; Good Response, 38% for 1a, and 38% for 1b. For single combination networks: Poor Response, 14% for 1a, and 29% for 1b; Good Response, 21% for 1a, and 27% of 1b. Hence HVR1 positions were approximately 6 times more likely to occur in these optimal networks than would be expected by chance.

Positions that coincided with hypervariable region 2 (HVR2, positions 473–480, 1% of E1E2 [23]) that also cover part of the first CD81 binding site, appeared only once in the 1a networks but relatively frequently in the 1b networks (Table S1). For multiple combinations in 1b: Poor Response, HVR2 positions appeared in 27% of 1b optimal networks; Good Response 23%. For single combinations in 1b: Poor Response, 9%; Good Response 4%.

Hypervariable region 3 (HVR3) extends from positions 431 to 466 [26] and covers approximately 7% of the E1E2 genes. This domain contained a relatively higher proportion of aa in the optimal networks. For multiple combinations: Poor Response, 19% for 1a, and 20% for 1b; Good Response, 18% for 1a, and 20% for 1b. For single combination networks: Poor Response, 7% for 1a, and 12% for 1b; Good Response, 9% for 1a, and 9% of 1b. Moreover 1b contained a greater number of different positions within this domain (Figure 4), whereas 1a had fewer but with position 434 being particularly prevalent and appearing 11 times in 1a networks and 7 in 1b networks.

Positions in the CD81 binding sites (473–493 (1), 524–536 (2a), 544–555 (2b), 613–622 (3), [23]–[25]) appeared only once in the optimal networks for CD81-2b and CD81-3, but were more frequent for domains 1 and 2a. In total for multiple combinations: Poor Response, 3% for 1a, and 20% for 1b; Good Response, 21% for 1a, and 25% for 1b. For single combination networks: Poor Response, 9% for 1a, and 18% for 1b; Good Response, 19% for 1a, and 10% of 1b.

Although the protein kinase PKR /eukaryotic translation initiation factor 2α phosphorylation homology domain (PePHD) has been implicated in failure of interferon therapy in HCV [15], none of the aa in this region (positions 659 to 670 [23]) appeared in any of the optimal networks regardless of Good or Poor response (Figure 4).

Some separating pairs appeared repeatedly within each set of networks. The most frequent of these, appearing 5 times, was the 531–626 (EI) separating pair for single combinations, Poor Response in 1a. This feature appeared in 6 of the nonresponders within the lowest cluster of the phylogenetic tree for genotype 1a (Figure 1). The most frequent pairs representing a Good Response among single combination networks were 431–481 (EE), 500–522 (QA) in 1a, and 407–434 (AQ) in 1b, each appearing 4 times. The most frequent of the individual positions appeared 7 times in their group for single combinations: 387 (poor response, 1a), 395, 481, 522 (good response 1a), and 407 (good response 1b).

## Discussion

Determining features in viral sequences that reflect likelihood of failure of antiviral therapy is a difficult problem. Simply by chance and the high variability of portions of these sequences, there will be collections of aa at some positions that will only appear in one response group. We instead based our analysis on aa pairs that were correlated within genotypes as these are more likely to indicate that evolutionary pressure has occurred at these positions related to viral fitness. Rather than describing all such networks we determined those that satisfied certain optimality criteria, reasoning that these were more likely to contain the most important features that reflect treatment outcome. The optimal networks and features we extract have 100% accuracy and 0% false discovery rate describing antiviral response and achieve this with minimal components. An alternative approach using Bayesian networks has also been employed to extract HCV genome features predicting response [27]. We found that therapy response groups could be separated with as few as 2 aa pairs if we allowed multiple aa combinations for these pairs, or with 5 pairs if only allowing a single aa combination for each pair. Under these optimality criteria there can be no fewer aa pairs that separate the groups. This implies that there is no single feature that can be used in each of these genotypes to predict therapy outcome, and probably indicates the many evolutionary pathways that HCV can take even within genotypes. This is particularly evident in genotype 1a where the phylogenetic tree splits into 3 main clusters, whereas genotype 1b sequences exhibit more random drift (Figure 1). It is unlikely these 1a clusters can be attributed to transmission within geographical sectors because the corresponding patients were mainly caucasian without any evidence of epidemiological links [16]. Thus the generation of the 3 genotype 1a clusters could be due to classical evolutionary forces linked to host-related immunity and viral fitness. However mode of transmission may play a part in these linkages. Genotype 1a in France has been associated with injecting drug users, while 1b is more prevalent among transfusion-associated cases [28]. This association was also observed in our HCV-infected cohort [16].

Further evidence of the different ways 1a and 1b viruses evolve are provided by the collection of the 50 aa pairs with the highest covariance (Figure 3). There were 9 separate subcomponents containing 58 positions as nodes with maximum node degree of 4 in the 1b network whereas the 1a collection formed a highly structured single component consisting of 18 nodes with maximum node degree of 11. This structure did not seem to determine therapy outcome however since SVR and NR individuals were scattered within these subgroups (Figure 1). Nevertheless our analysis did pick out some pairs that were present in responders versus nonresponders within some of these groups. For instance the 531–626 (EI) separating pair was indicative of a poor response to this therapy for one of the 1a subgroups. Each of these positions was highly prevalent in the optimal networks with position 531 (SVR:A, NR:E) occurring 13 different times and position 626 occurring 11 times (SVR:V, NR:I). These positions occurred infrequently for 1b: 531 twice and 626 once. The 1a nonresponders who expressed the 531–626 (EI) pair or the 3 SVR individuals with an A at position 531 are all grouped in the lower 1a cluster in Figure 1. This group of 14 individuals is identified by the ATLESA or TTM(E/A)SA sequences at positions 216, 243, 456, 655, 686 and 710 in the network in Figure 3A. It is of interest that the calculations of minimal networks has extracted aa features for this subgroup that determines within this group what is indicative of therapy failure or success. So although a single feature cannot predict treatment outcome, within this phylogenetic cluster a single feature does exist. Position 531 is situated in the 2a CD81 binding site and is adjacent to the glycosylation site at position 532. Similarly position 626 is next to the glycosylation site that is coded by 623–625. A subtle change in aa composition of HCV envelope glycoproteins has been demonstrated to modify their immunity and/or viral entry efficiency [12], [16], [29]. It can be hypothesized that the presence of a negatively charged E at 531 *versus* the hydrophobic A could influence HCV immunity or the entry step into hepatocytes.

HVR2 was much more likely to appear in 1b networks than would occur by chance, whereas it appeared only once in the 1a networks. The other hypervariable regions were also highly prevalent in the networks but this did not differ between genotypes. Except for a single occurrence, only the CD81 binding regions 1 and 2a appeared in the networks, where region 2a overlaps with HVR2. Although the PePHD domain has been implicated in the failure of interferon therapy none of these positions appeared in these networks.

There are several limitations to our methods. One of these is that the calculation of the optimal separating set determines one such optimal set for each problem where there may be a number of optimal solutions. Hence our method does not necessarily extract all the pairs that may influence response to therapy and so these are only subnetworks of the important positions and pairs. Furthermore only the dominant sequence in an individual's viral quasispecies was determined. Viral clones that are present at low frequency before the commencement of therapy may play a major role in its failure, and our analysis did not incorporate this aspect. Deep sequencing may uncover greater information on which to base predictions of treatment response, but would certainly complicate the analysis.

In summary, this approach of determining optimal sets of covarying pairs has identified regions of particular importance to differentiating an individual's response to antiviral therapy for HCV. It has also quantified the difficulty in achieving response and the different pathways the virus can take to evade clearance given that there were no single pairs that completely separated response groups. No fewer than 5 aa pairs were required to separate response groups when allowing single combinations for each pair. The optimal covarying networks determined here have extracted regions for further in vitro investigation.

## Supporting Information

Table S1 **Covarying pairs in optimal networks.**
(DOCX)
