# Supplementary material for: Genotype 1 Hepatitis C Virus Envelope Features That Determine Antiviral Response Assessed through Optimal Covariance Networks
Source: PLoS One. 2013 Jun 20;8(6):e67254. doi: 10.1371/journal.pone.0067254 (PMC3688619; doi:10.1371/journal.pone.0067254)
Supplement: Table S1 — Covarying pairs in optimal networks. (DOCX) [file pone.0067254.s001.docx]

# Genotype 1 hepatitis C virus envelope features that determine antiviral response assessed through optimal covariance networks

# John M. Murray^1,2^, Rémy Moenne-Loccoz^3^, Aurélie Velay^4,5^, François Habersetzer^3,6,7^, Michel Doffoël^3,6,7^, Jean-Pierre Gut^7^, Isabel Fofana^6,7^, Mirjam B. Zeisel^6,7^, Françoise Stoll-Keller^6,7,8^, Thomas F. Baumert^3,6,7^, Evelyne Schvoerer^4,5^

## ^1^School of Mathematics and Statistics, University of New South Wales, Sydney, NSW, Australia; ^2^The Kirby Institute, University of New South Wales, Sydney, NSW, Australia; ^3^Pôle Hépato-digestif, Hôpitaux Universitaires de Strasbourg, Nouvel Hôpital Civil, Strasbourg, France; ^4^Université de Lorraine, EA Stress Immunité et Pathogènes, Vandoeuvre-les-Nancy, France; ^5^CHU Nancy, Pôle Laboratoires, Virologie, Vandoeuvre-les-Nancy, France; ^6^Institut National de la Santé et de la Recherche Médicale (Inserm), U748, Strasbourg, France; ^7^Université de Strasbourg, Strasbourg, France; ^8^Laboratoire de Virologie, Hôpitaux Universitaires de Strasbourg, Strasbourg, France

Table S1: Covarying pairs in optimal networks. Covarying pairs and features found in NR but not SVR (Poor Response), or found in SVR but not NR (Good Response). The Separating Set describes the group over which the covarying pairs were calculated. Minimal networks based on the fewest number of edges are described in the rows where the Prob column displays No, whereas rows where this is Yes describe minimal networks that incorporate the level of covariance of each edge. When only a single separating pair can be chosen between nodes then the Single Feature column displays Yes. The positions refer to aa positions in the HCV genome for which E1E2 commences at position 192. n specifies the number of individuals that exhibit that separating pair(s), and p describes the weight of the pair according to the calculation of $\bar{S}-S$, where $\bar{S}$ is the maximum over all covarying pairs for that group (hence a smaller value is better).

| Geno | Sep. Set | Prob | Single Feature | Poor Response | | Good Response | |
| --- | --- | --- | --- | --- | --- | --- | --- |
|  |  |  |  | Pairs (n, p) | Features | Pairs (n, p) | Features |
| 1a | All | No | No | 223-391(11)  397-522(19)  414-416(2) |  | 386-531(7)  397-522(13)  522-531(5) |  |
|  |  | No | Yes | 223-393(5)  373-434(8)  387-610(5)  414-442(3)  531-626(6)  558-655(5) | AA  VD  TD  VL  EI  TD | 198-522(1)  500-522(5)  223-531(3)  384-395(2)  395-560(4)  434-720(3) | SM  QA  TA  ET  NY  NI |
|  |  | Yes | No | 373-610(10, 6.3)  397-522(19, 6.5)  655-710(3, 1.4) |  | 340-481(2, 1.3)  481-558(2, 0.1)  384-531(11,6.4)  434-531(9, 6.0) |  |
|  |  | Yes | Yes | 219-223(6, 3.5)  373-434(8, 6.5)  387-610(5, 6.2)  414-442(3, 6.5)  481-558(2, 0.1)  531-626(6, 5.9) | AA  VD  TD  VL  ET  EI | 223-626(3, 4.8)  395-560(4, 6.2)  431-481(3, 3.9)  481-558(1, 0.1)  500-522(5, 6.3)  531-655(3, 6.1) | TV  NY  EE  DS  QA  AE |
|  | SVR | No | No | 384-404(17)  391-626(13)  463-580(11) |  | 396-434(6)  399-524(9)  407-653(4) |  |
|  |  | No | Yes | 223-330(3)  241-501(2)  241-610(7)  373-434(8)  387-521(7)  531-626(6) | AA  PS  AD  VD  VK  EI | 198-395(2)  391-653(4)  399-524(7)  401-522(6)  431-481(3) | SN  TN  LV  SA  EE |
|  |  | Yes | No | 223-391(11, 1.4)  384-404(17, 2.4)  473-626(6, 2.0) |  | 391-653(7, 2.1)  404-580(8, 2.2)  405-560(7, 1.9) |  |
|  |  | Yes | Yes | 219-223(6, 1.1)  219-655(1, 1.2)  373-434(8, 2.1)  387-521(7, 1.9)  387-610(5, 2.2)  531-626(6, 2.5) | AA  TH  VD  VK  TD  EI | 216-391(5, 1.4)  395-560(4, 2.2)  401-522(6, 2.4)  431-481(3, 2.3)  481-558(1, 1.3) | AT  NY  SA  EE  DS |
|  | NR | No | No | 303-394(7)  397-522(19)  434-626(10) |  | 397-522(13)  399-434(11) |  |
|  |  | No | Yes | 387-610(5)  399-438(5)  401-407(4)  463-626(6)  501-622(6)  558-655(5) | TD  FL  GP  TI  TI  TD | 198-531(1)  223-531(3)  395-463(4)  410-603(2)  434-720(3)  500-522(5) | TV  TA  NA  KI  NI  QA |
|  |  | Yes | No | 223-340(6, 2.6)  394-466(10, 4.5)  397-522(19, 4.4) |  | 397-522(13, 4.4)  399-434(11, 4.1) |  |
|  |  | Yes | Yes | 223-340(5, 2.6)  243-558(4, 2.7)  387-558(1, 1.9)  308-330(6, 3.5)  401-407(4, 4.4)  431-610(4, 3.7)  531-626(6, 3.8) | AI  AT  IS  MA  GP  AD  EI | 395-463(4, 4.5)  431-481(3, 2.3)  481-558(1, 1.7)  500-522(5, 4.3)  531-655(3, 3.8)  626-655(3, 3.5) | NA  EE  DS  QA  AE  VE |

| 1b | All | No | No | 384-478(19)  444-475(11) |  | 384-478(17)  404-405(12)  477-712(11) |  |
| --- | --- | --- | --- | --- | --- | --- | --- |
|  |  | No | Yes | 233-528(2)  253-398(4)  398-608(6)  392-641(5)  395-407(2)  408-531(4)  479-580(2) | GR  IG  SL  QN  GA  QE  SL | 313-625(5)  386-387(3)  396-713(2)  402-500(4)  438-444(3)  446-464(5)  478-705(2)  522-705(4) | IS  TV  VA  FA  IR  RE  DV  SV |
|  |  | Yes | No | 384-444(12, 2.4)  384-478(19, 2.4) |  | 397-404(12, 2.0)  477-712(11, 2.3)  478-492(11, 2.0) |  |
|  |  | Yes | Yes | 251-397(2, 2.0)  285-596(4, 2.3)  313-391(6, 1.9)  386-574(4, 2.0)  438-444(2, 1.7)  476-528(4, 2.4)  501-533(2, 1.8)  705-712(1, 0.3) | IR  LR  VV  RA  IS  ER  EA  IC | 233-388(5, 2.4)  251-446(5, 2.2)  330-492(2, 1.2)  386-387(3, 2.1)  407-438(4, 2.3)  438-444(3, 1.7)  528-641(3, 2.0)  705-712(2, 0.3) | NS  TR  AK  TV  SI  IR  TN  VV |
|  | SVR | No | No | 384-444(12)  384-478(19) |  | 391-712(13)  398-477(16)  405-641(14) |  |
|  |  | No | Yes | 197-444(1)  200-500(4)  387-404(3)  391-395(5)  392-641(5)  398-464(4)  398-653(3)  475-476(5) | VI  AS  TT  VA  QN  SK  TD  TE | 299-641(4)  313-466(5)  387-479(5)  391-712(5)  395-626(3)  407-434(6) | ET  ID  VN  TL  AV  AQ |
|  |  | Yes | No | 384-478(19, 1.3)  444-475(11, 1.1) |  | 251-392(13, 1.2)  398-477(16, 1.3)  407-434(9, 1.0) |  |
|  |  | Yes | Yes | 233-528(2, 1.4)  313-391(6, 1.3)  313-625(2, 0.6)  386-434(3, 1.0)  398-501(5, 1.3)  445-501(6, 1.1)  445-708(1, 0.7)  444-449(3, 0.9)  533-608(1, 0.2) | GR  VV  LS  RQ  SQ  HQ  KA  VA  AL | 251-524(3, 0.9)  271-580(6, 1.2)  299-313(3, 1.0)  407-434(6, 1.0)  466-712(3, 0.9)  533-608(4, 0.2)  610-710(4, 0.9) | TA  LT  QI  AQ  AL  KM  HA |
|  | NR | No | No | 251-478(14)  386-478(14)  501-574(9) |  | 251-478(15)  384-574(10)  397-404(12)  477-712(11) |  |
|  |  | No | Yes | 253-445(4)  391-396(6)  404-479(5)  454-531(2)  477-528(3)  596-674(5) | IR  VT  TG  EG  SR  RI | 268-386(4)  395-407(3)  407-434(6)  398-445(4)  399-708(5)  522-705(4)  531-641(4) | AT  AS  AQ  GN  LV  SV  ET |
|  |  | Yes | No | 256-391(13, 1.5)  285-480(2, 0.2)  398-464(13, 1.3)  574-653(3, 0) |  | 197-405(6, 0.7)  397-712(12, 1.3)  407-479(14, 0.9)  574-653(4, 0) |  |
|  |  | Yes | Yes | 257-434(4, 1.4)  285-480(1, 0.2)  392-705(7, 1.2)  398-608(6, 0.8)  400-414(4, 0.8)  404-479(5, 1.0)  574-653(1, 0) | AN  FW  QI  SL  AV  TG  IN | 233-387(5, 0.6)  235-674(2, 0.8)  394-674(5, 1.0)  400-674(3, 0.8)  285-480(2, 0.2)  407-434(6, 1.4)  407-479(2, 0.9)  501-610(2, 0.6)  574-653(1, 0) | GV  TV  YI  VI  FQ  AQ  SS  SD  AD |
